# National diabetes registries: do they make a difference?

**DOI:** 10.1007/s00592-020-01576-8

**Published:** 2020-08-08

**Authors:** Jessica C. G. Bak, Erik H. Serné, Mark H. H. Kramer, Max Nieuwdorp, Carianne L. Verheugt

**Affiliations:** Department of Vascular Medicine, Amsterdam UMC, Location AMC, Meibergdreef 9, 1105 AZ Amsterdam, The Netherlands

## Abstract

**Aims:**

The global epidemic of diabetes mellitus continues to expand, including its large impact on national health care. Measuring diabetes outcomes and their causes of variation highlights areas for improvement in care and efficiency gains; large registries carry this potential. By means of a systematic review, we aimed to give an overview of national registries worldwide by quantifying their data and assessing their influence on diabetes care.

**Methods:**

The literature on MEDLINE up to March 31, 2020, was searched, using keywords diabetes mellitus, national, registry, registration, and/or database. National disease-specific registries from corresponding articles were included. Database characteristics and clinical variables were obtained. All registries were compared to the ICHOM standard set of outcomes.

**Results:**

We identified 12 national clinical diabetes registries, comprising a total of 7,181,356 diabetic patients worldwide. Nearly all registries recorded weight, HbA1c, lipid profile, and insulin treatment; the recording of other variables varied to a great extent. Overall, registries corresponded fairly well with the ICHOM set. Most registries proved to monitor and improve the quality of diabetes care using guidelines as a benchmark. The effects on national healthcare policy were more variable and often less clear.

**Conclusions:**

National diabetes registries confer clear insights into diagnostics, complications, and treatment. The extent to which registries influenced national healthcare policy was less clear. A globally implemented standard outcome set has the potential to improve concordance between national registries, enhance the comparison and exchange of diabetes outcomes, and allocate resources and interventions where most needed.

## Introduction

Diabetes mellitus presents a significant burden in Europe; the IDF Diabetes Atlas 2017 estimates that it affects 58 million people and costs a staggering 145 billion euros annually [1]. Its prevalence is expected to rise even further in the future as a result of rising obesity and increased unhealthy lifestyles, as well as an aging population [2]. There is an urgent need to identify ways to improve outcomes for those who have diabetes. Measuring and comparing diabetes outcomes—and identifying the causes of variation—help to highlight areas where better outcomes and efficiency gains can be achieved. Registries have the potential to collect large datasets that can inform decision-making [3]. They can act as a tool for local quality control and benchmarking against national treatment aims, and also assess the value of therapies and treatment models that work in clinical practice and deliver for people with diabetes and insurance payers, in addition to randomized controlled trials [4–7].

As a response to the demands of the St Vincent Declaration for quality assurance in diabetes care [8], several countries initiated some kind of diabetes registry. Data from the IDF demonstrate that there has been an absolute increase in the number of countries with some kind of diabetes registry—from 23 in 2011 to 30 in 2014 (out of 47 countries)—however, more than 83% were considered to be incomplete [1]. Most countries have civil or vital statistics registration systems, and all national health information systems routinely collect mortality data. Nevertheless, these general registration systems are unable to consistently provide information on monitoring and managing diabetes. In addition, standardized outcome definitions and common methods of data collection are essential to outcome comparison and subsequent improvements. The International Consortium on Health Outcome Measurement (ICHOM) is leading the way by systematically engaging with clinical experts and patients to find consensus on outcomes that matter to patients [9]. In 2018, ICHOM published the first standard set of outcomes for adults with type 1 and type 2 diabetes [10]. Despite the awareness on the potential of national diabetes databases [1], little is known about the structure and usefulness of national diabetes databases in daily practice.

By means of a systematic review, this study aims to give an overview of the national registries of diabetes mellitus around the world by quantifying their data and assessing their influence on diabetes care.

## Methods

### Search strategy and study selection

A literature search in the MEDLINE database was performed to identify studies that used national data sets of diabetes mellitus patients as a data source. The search was performed on April 1, 2020; publications up until March 31, 2020, were included. The MEDLINE database was systematically searched for the following query terms in the title and abstract field: diabetes mellitus AND national AND (registry OR registration OR database). For reasons of clarity, the search was limited to articles written in the English language. The following inclusion criteria were used: (a) articles describing registries or databases; (b) collecting prospective data on type 1 or type 2 diabetic patients; (c) aiming to ultimately include all diabetic patients within one country; (d) a minimum of 1000 included patients. Studies were excluded if describing databases or registrations that are: solely regional, cross-continental, or combining multiple countries; registering primary care only; a select patient population established for cohort studies or trials; set up originally for monitoring other populations than diabetes mellitus; not covering the full diabetic population (narrowed to specific subgroups or less than 1,000 patients included); linkage of multiple clinical or non-clinical registries creating a database not intending to follow-up of the diabetic patient population for a longer period of time; mainly or only including children; currently no longer actively in use; information on current status of the registry could not be provided or validated by administrators of the registry; information on current status of the registry could not be provided or validated by administrators of the registry.

### Data extraction and analysis

All abstracts, or full-text articles if abstracts provided too little information, were separately reviewed by two researchers (J.B. and C.V.). Articles were selected according to the PRISMA (preferred reporting items for systematic reviews and meta-analyses) statement [7]. Identified articles were entered in Mendeley Desktop (Version 1.19.4; Glyph & Cog). After removal of excluded articles, articles were categorized by national registry. Data about the database features and registered characteristics of patients enrolled in the database (patient-specific and disease characteristics, data about diagnostic methods used, and treatment received) were collected. Information was also retrieved from the original websites of the registries and annual reports published on the  internet using data of the registries. Collected data were entered in a database in Microsoft Excel 2016. Administrators of all registries were contacted to validate the data collected in their registry and to provide information about current numbers of patients included in the database. Definitions of various variables varied per registry. Severe hypoglycemia was defined as a glucose below 54 mg/dl (3.0 mmol/l) or hypoglycemia for which help from outside sources or hospital admission was needed, or hypoglycemia accompanied by loss of consciousness or seizures. PROMs describe health outcome measures from the perspective of patients, enabling to analyze the treatment effect on quality of life, well-being, and other health outcomes. Lifestyle management is characterized as patient education focused on lifestyle modifications. Diet was defined as low caloric food intake as a treatment for diabetes. Glucose sensing comprised both real-time continuous glucose monitoring (RT-CGM) and flash glucose monitoring (FGM). Other medication than antidiabetic medication was characterized as drugs used for treatment of diseases associated with diabetes mellitus, complications, or comorbidities such as lipid-lowering drugs, antihypertensive drugs, and medication treating neuropathic pain.

Data on the specific goals of the registry, what data sources were used, and which outcome measures were registered, were collected from published articles, reports, and registry websites. In addition, we compared the set of registered variables of all national diabetes registries with the standard set of the International Consortium for Health Outcome Measures (ICHOM) [10]. The ICHOM Standard Set for Adults with type 1 and 2 Diabetes consists of 13 health outcomes, which can be subdivided in 6 domains: diabetes control (glycemic control), patient-reported outcomes (psychological well-being, diabetes distress, depression), acute events (diabetic ketoacidosis, hyperglycemic hyperosmolar syndrome, hypoglycemia), chronic complications (micro- and macrovascular complications, nervous system complications, treatment complications), survival, health services (financial barriers to treatment, healthcare utilization). Furthermore, to enable the comparison of outcomes ICHOM defines case-mix variables: demographic factors (sex, year of birth, ethnicity, educational level), diagnosis profile (diabetes type, year of diagnosis, comorbidities), lifestyle and social factors (smoking, alcohol frequency and amount, physical activity, social support), treatment factors (diabetes treatment, lipid-lowering therapy, treatment adherence to dietary advice, exercise, blood sugar monitoring, and medication) [9]. Furthermore, numbers of included patients were collected from articles or through direct contact with registries. The estimated prevalence of the total diabetes population per country was calculated, dividing the number of included patients in the registry by the total number of diabetic patients in the corresponding country. The total number of diabetic patients of each country was gathered from the website of the World Health Organisation or national diabetes foundations [11]. For every registry, their ability of to capitalize on the value of registries was assessed. This was defined as the ability from a registry to support improvement in the quality of care and adherence to treatment guidelines, the use of registry data in epidemical research, and if their data are used for healthcare policy making. This is based on published data from the registries on these subjects. Use of data for healthcare policy making is defined as published data on treatment suggestions. Data quality is defined as comparability (extent to which coding, classification procedures, and definition are according to international guidelines), completeness (the degree of availability of the required data), validity (accuracy of the data), and timeliness (time expectation for accessibility and availability the data) [12]. Information on data quality and data quality assurance is gathered from published data from the registries. We reported the systematic review according to the PRISMA [11].

## Results

### Study selection

**Fig. 1:**
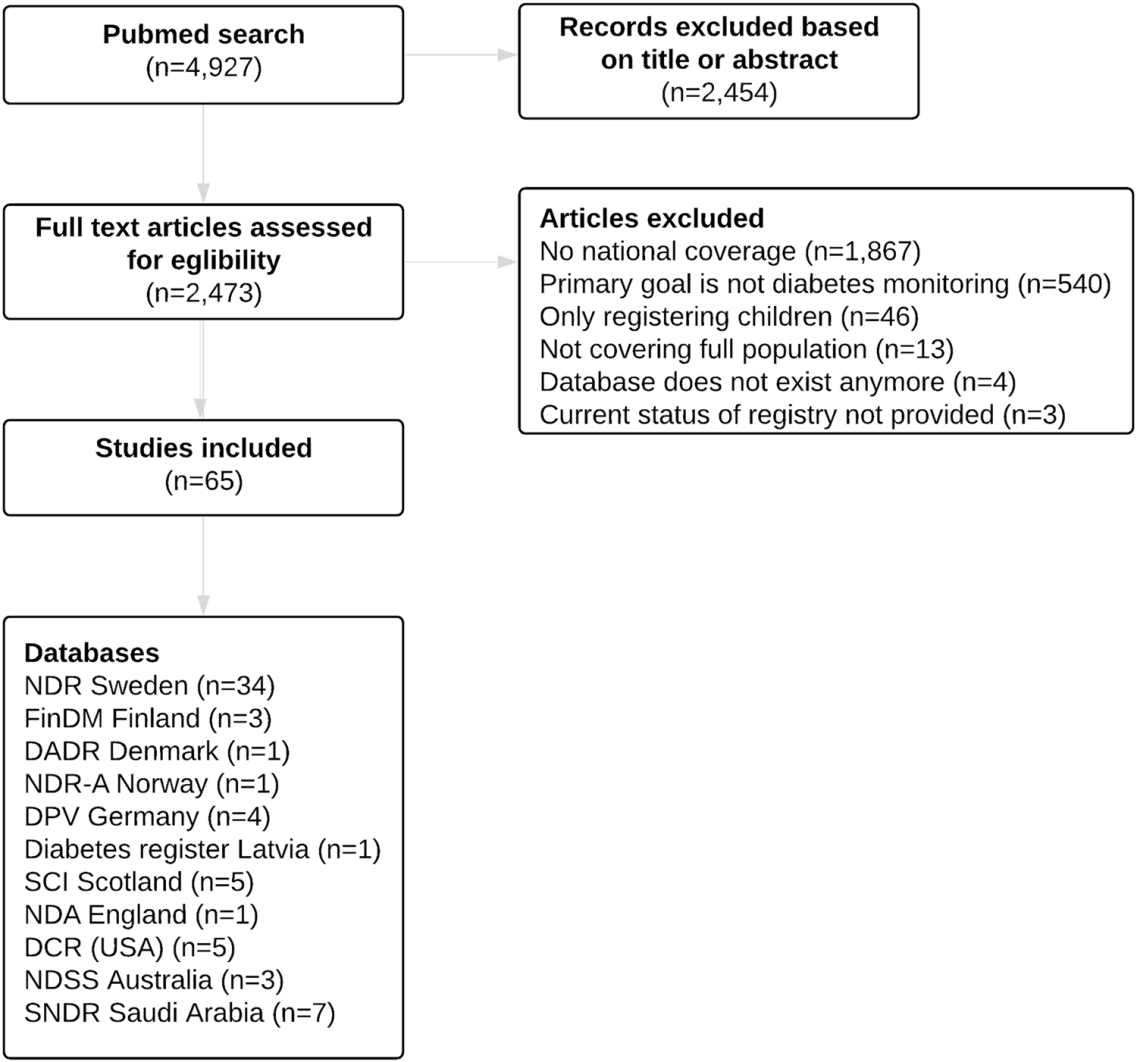
Flowchart on study selection by database

The literature search yielded 4,927 articles as displayed in Fig. 1. After excluding articles based on title or abstract, a total of 2,473 articles were eligible for assessment. Other 2,408 articles were excluded based on pre-specified inclusion criteria, rendering 65 articles for review.

### National diabetes registries

**Fig. 2:**
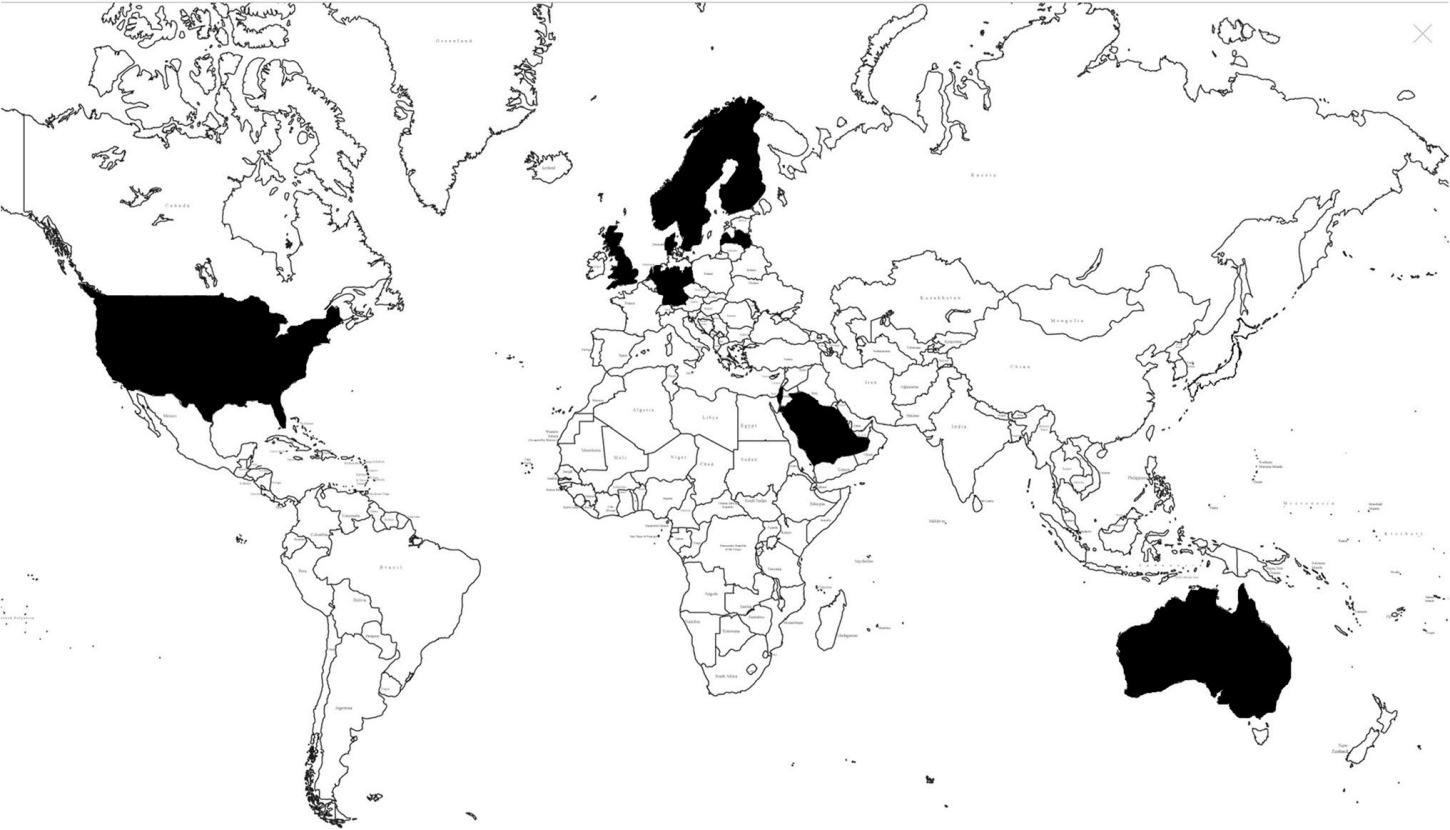
World map of nations with national clinical diabetes registries indicated in black

Worldwide, 12 national clinical diabetes databases were identified originating from Sweden (National Diabetes Register; NDR), Finland (Diabetes in Finland; FinDM), Denmark (Danish Adult Diabetes Registry; DADR), Norway (Norwegian Diabetes Register for Adults; NDR-A), The Netherlands (Dutch Pediatric and Adult Registry of Diabetes; DPARD), Germany (Diabetes-Patienten-Verlaufsdokumentation; DPV), Scotland (Scottish Care Information-Diabetes; SCI-Diabetes), Latvia (Diabetes Register), England (National Diabetes Audit; NDA), the USA (Diabetes Collaborative Registry; DCR), Australia (National Diabetes Services Scheme; NDSS), Saudi Arabia (Saudi National Diabetes Registry; SNDR). Figure 2 shows the countries involved, spread over four different continents.

Table 1 gives an overview of the national registries and their characteristics displayed by country and corresponding registry. All databases together accounted for a total of 7,181,356 diabetic patients. The NDA of England holds almost half of the patients included. The SCI-Diabetes database covers the largest part of the national Scottish population of diabetic patients, showing complete coverage. The Scandinavian countries follow with coverages up to 95%. Among databases worldwide, the NDSS of Australia is the first national diabetes registry established, whereas the Dutch DPARD is the registry most recently founded. Most registrations register patients treated in both primary and secondary or tertiary care. Some registries have not included children, whose data are collected in a separate pediatric national diabetes database. All national clinical diabetes registries comprised patients with type 1 and type 2 diabetes mellitus.

**Table 1:** Overview of national clinical diabetes registries throughout the world

National diabetes registry	Registry founded	Number of included patients	Estimated coverage of total diabetes population	Primary care	Secondary/ tertiary care	Children	Type 1 DM	Type 2 DM
Sweden (NDR)	1996	444,648	93%	+	+	+	+	+
Finland (FinDM)	1997	455,261	95%	+	+	+	+	+
Denmark (DADR)	2004	30,000	12%	+	+	–	+	+
Norway (NDR-A)	2005	60,000	24%	+	+	–	+	+
The Netherlands (DPARD)	2017	20,087	2%	−	+	+	+	+
Germany (DPV)	2000	550,000	8%	+	+	+	+	+
Latvia (Diabetes Register)	1997	91,571	90%	+	+	+	+	+
Scotland (SCI-Diabetes)	2000	298,504	100%	+	+	+	+	+
England (NDA)	2004	3,200,185	68%	+	+	−	+	+
USA (DCR)	2014	1,000,000	3%	+	+	−	+	+
Australia (NDSS)	1987	1,324,948	80 – 90%	+	+	+	+	+
Saudi Arabia (SNDR)	2001	150,000	2%	+	+	+	+	+

*NDR* National Diabetes Register,* FinDM* Diabetes in Finland,* DADR* Danish Adult Diabetes Registry,* NDR-A* Norwegian Diabetes Register for Adults,* DPARD* Dutch Pediatric and Adult Registry of Diabetes,* DPV* Diabetes-Patienten-Verlaufsdokumentation,* SCI-Diabetes* Scottish Care Information-Diabetes,* NDA* National Diabetes Audit,* DCR* Diabetes Collaborative Registry,* NDSS* National Diabetes Services Scheme,* SNDR* Saudi National Diabetes Registry,* DM* diabetes mellitus

### Goals

National diabetes registries have been founded with different goals in mind. Most registries were primarily started as a tool for local quality control and benchmarking against the national treatment aims as defined in guidelines [13–22]. The main focus is on improving patient care and education. Sweden, Denmark, Norway, The Netherlands, Germany, England, and the USA provided the assessment and improvement in quality of diabetes care as one of their primary purposes [13, 15–18, 21, 23]. The NDSS of Australia is the only registry established to enhance the self-management in patients with diabetes and to provide support and subsidized diabetes products [24]. Research is mentioned as a secondary aim by Sweden, Finland, Norway, Denmark, The Netherlands, Germany, Latvia, the USA, and Saudi Arabia [14–19, 21, 22]. Specifically, epidemiological research can provide data on time trends and variations (prevalence, incidence and mortality) by age, gender, demographic, and clinical characteristics. Subsequently, as a third aim, these data can be used as a surveillance instrument to support healthcare policy, such as evaluation of the (direct and indirect) economic effects of diabetes or evaluating prevention programs or specific therapeutic interventions. Sweden, Norway, Scotland, England, the USA, and Saudi Arabia have proposed this as one of their goals [16, 20, 22, 25–27].

### Data collection

Various ways of collecting data exist: by manual entry, via automated data capture from electronic health records, or by linkage of different national databases or quality registries. Most registries use a combination of methods to collect their data. Sweden, Denmark, Norway, The Netherlands, Germany, England, USA use automated transmission from electronic health records as one of the data collection methods used [15, 16, 18, 23, 28, 29]. Depending on the primary goal, data are collected from different data sources. Hospital data sources provide details with respect to clinical and laboratory data and education, whereas national administrative data sources are often necessary to obtain data on mortality or to obtain data on time trends. Sweden, Finland, Denmark, Norway, Latvia, Scotland, England and Saudi Arabia make use of data linkage to other databases enabling information exchange [14–16, 19, 20, 23, 28, 30]. Each database renders an unique patient identifying variable, enabling patient follow-up over time and potential linkage of databases and registries. Variables used as a patient identifier differ among registries, varying from in-hospital patient identification numbers, national identification numbers, national insurance numbers, national health service numbers to patient keys generated by the registries. Every registry used assigned identification numbers for every patient, allowing for follow-up over a long period of time.

### Outcome measures

Variables recorded in diabetes registries can be subdivided into parameters describing the process of clinical care (comprising physical examination, diagnostics and delivered treatment) and describing outcome measures (i.e., glycemic regulation, blood pressure control, PROMs, complications, and mortality). Some variables may be used as both process and outcome parameters. The variable HbA1c, for instance, can be used as a process parameter signaling quality of care or as a surrogate outcome parameter of glycemic control.

Table 2 shows a detailed outline of process parameters, comprising patient features, diagnostic parameters, disease characteristics recorded by registry, and whether these match with the standard ICHOM set. Variables are roughly divided in the following domains, i.e., demographic factors (gender and ethnicity), diagnosis profile (date of diagnosis, eye and foot examination, kidney function, autoantibodies), lifestyle and social factors (smoking status, height, weight, body mass index), diabetes control (HbA1c, blood pressure, lipid profile, liver enzymes), complications (microvascular organ damage and severe hypoglycemia), mortality, and treatment factors (lifestyle management, diet, oral medication, insulin treatment and glucose sensing). Among physical examination parameters, all databases register variables concerning body mass index or weight and height. Blood pressure is not consistently registered. Moreover, considerable variation among registries exists in laboratory parameters recorded. HbA1c and lipid profile are included in all registries but one. Other laboratory variables are registered by fewer registries, such as glucose level at diagnosis and liver enzymes. The Netherlands, Scotland, and Germany record autoantibodies associated with diabetes mellitus, anti-glutamic acid decarboxylase (GAD), islet antigen 2 (IA2), and islet cell (ICA) antibodies.

**Table 2:** Overview of process and outcome parameters reported by national diabetes registries and ICHOM

Parameters	Sweden (NDR)	Finland (FinDM)	Denmark (DADR)	Norway (NDR-A)	The Netherlands (DPARD)	Germany (DPV)	Latvia (Diabetes Register)	Scotland (SCI-Diabetes)	England (NDA)	USA (DCR)	Australia (NDSS)	Saudi Arabia (SNDR)	ICHOM
**Demographic factors**													
Gender	+	+	+	+	+	+	+	+	+	+	+	+	+
Ethnicity	−	+	−	+	+	−	−	+	+	+	−	−	+
**Diagnosis profile**													
Date or year of diagnosis	+	+	+	+	+	+	+	+	+	+	+	+	+
Eye examination	+	+	+	+	+	+	+	+	+	+	−	−	+
Foot examination	+	−	+	+	+	+	+	+	+	+	−	−	+
Creatinine and/or eGFR	+	−	+	+	+	+	+	+	+	+	−	+	+
Urine (micro-)albumin	+	+	+	+	+	+	+	+	+	+	−	+	+
GAD,IA2, or ICA antibodies	−	−	−	−	+	+	−	+	−	−	−	−	−
MODY	−	−	−	−	+	+	−	+	−	−	−	−	−
**Lifestyle and social factors**													
Smoking status	+	+	+	+	+	+	−	+	+	+	−	+	+
Weight and height or BMI	+	+	+	+	+	+	+	+	+	+	+	+	+
**Diabetes control**													
HbA1c at time of diagnosis	−	−	−	−	+	+	+	−	−	−	−	−	−
HbA1c at follow-up	+	+	+	+	+	+	+	+	+	+	−	+	+
Glucose at time of diagnosis	−	+	−	−	+	+	+	−	−	−	−	−	−
Random blood glucose test	−	+	−	−	−	+	−	+	−	+	−	+	−
Blood pressure	+	−	+	+	+	+	+	+	+	+	−	−	+
Lipid profile	+	+	+	+	+	+	+	+	+	+	−	+	+
Liver enzymes	−	−	−	−	−	+	−	+	−	+	−	−	−

*ICHOM* International Consortium for Health Outcome Measures,* NDR* National Diabetes Register,* FinDM* Diabetes in Finland, * DADR* Danish Adult Diabetes Registry,* NDR-A* Norwegian Diabetes Register for Adults,* DPARD* Dutch Pediatric and Adult Registry of Diabetes,* DPV* Diabetes-Patienten-Verlaufsdokumentation,* SCI-Diabetes* Scottish Care Information-Diabetes,* NDA* National Diabetes Audit,* DCR* Diabetes Collaborative Registry,* NDSS* National Diabetes Services Scheme,* SNDR* Saudi National Diabetes Registry

Table 3 shows an overview of diabetic events, microvascular and macrovascular complications, PROMs, and treatment variables by registry, most of which are viewed as outcome parameters. Macrovascular complications are not consistently registered among all registries. All-cause mortality is recorded in the registries of Finland, Germany, Scotland, USA and Saudi Arabia. At the moment, Sweden and Germany have the only registries that systematically record patient-reported outcome measures (PROMs). Insulin treatment is included throughout all registries; oral antidiabetic medication in some. Dietary and lifestyle treatment are not recorded in most databases. Registries founded more recently have included the parameters glucose sensing and lifestyle treatment more often. The registries of USA and Scotland resemble the variables recommended by ICHOM to the greatest extent.

**Table 3:** Overview of diabetic microvascular and macrovascular complications, survival, PROMs, and treatment specifics reported by national diabetes registries and ICHOM

Parameters	Sweden (NDR)	Finland (FinDM)	Denmark (DADR)	Norway (NDR-A)	The Netherlands (DPARD)	Germany (DPV)	Latvia (Diabetes Register)	Scotland (SCI-Diabetes)	England (NDA)	USA (DCR)	Australia (NDSS)	Saudi Arabia (SNDR)	ICHOM
**Acute events**													
Severe hypoglycemia	+	+	+	+	−	+	+	+	+	−	−	−	+
**Microvascular and macrovascular complications**													
Diabetic retinopathy	+	+	−	+	−	+	+	+	−	+	−	+	+
Diabetic nephropathy	+	+	−	+	+	+	+	+	+	+	−	+	+
Diabetic neuropathy	−	+	−	+	−	+	+	+	−	+	−	+	+
Peripheral arterial disease, ulcus cruris, or lower limb amputation	−	+	−	+	−	+	+	+	−	+	−	+	+
Cardiac disease	−	+	−	+	+	+	+	+	−	+	−	−	+
**Survival**													
Cardiac mortality	−	−	−	−	−	−	−	−	−	−	−	−	+
All-cause mortality	−	+	−	−	−	+	+	+	−	+	−	+	+
**PROMs**													
PROMs	+	−	−	−	−	+	−	−	−	−	−	−	+
**Treatment factors**													
Lifestyle management	−	−	−	−	+	+	−	+	+	+	−	−	+
Diet as treatment	+	−	−	+	−	+	+	−	−	+	+	+	+
Oral antidiabetic medication	+	+	+	+	+	+	+	+	−	+	+	+	+
Insulin treatment	+	+	+	+	+	+	+	+	+	+	+	+	+
Method of administration insulin (pump or pen)	+	+	+	+	+	+	−	+	+	+	+	−	+
FGM/CGM	−	−	−	+	+	+	−	+	−	+	−	−	−
Other medication than antidiabetic medication	+	−	+	+	+	+	−	+	−	+	−	−	−

*ICHOM * International Consortium for Health Outcome Measures,* PROMs* patient-reported outcome measures,* NDR* National Diabetes Register,* FinDM* Diabetes in Finland,* DADR* Danish Adult Diabetes Registry,* NDR-A* Norwegian Diabetes Register for Adults,* DPARD* Dutch Pediatric and Adult Registry of Diabetes,* DPV* Diabetes-Patienten-Verlaufsdokumentation, *SCI-Diabetes* Scottish Care Information-Diabetes, *NDA* National Diabetes Audit, *DCR* Diabetes Collaborative Registry, *NDSS* National Diabetes Services Scheme, *SNDR* Saudi National Diabetes Registry,* FGM* flash glucose monitoring,* CGM* continuous glucose monitoring

### The ability to capitalize on the value of registries

The main purpose of many diabetes registries is to support improvement in the quality of care and adherence to national and international treatment guidelines. Reports or studies from Sweden, Denmark, Norway, Germany, Scotland, England, and the USA have shown to effectuate this [15, 16, 18, 23, 28, 29, 31–35]. Treatment targets from national guidelines were achieved in a great part of the diabetic population [26, 28, 36]. However, in specific subgroups treatment goals were not reached. Almost all national registries were able to identify unmet treatment targets for HbA1c, blood pressure, or lipid levels [16, 17, 28, 31, 36–42]. Data from the Norwegian diabetes registry showed that 10% of adult patients with type 1 diabetes treated in specialist clinics reached treatment goals for HbA1c, LDL cholesterol, and blood pressure [31]. Another purpose of diabetes registries conducting epidemiological research to give insight in incidence, prevalence, or complications. Data from registries from Sweden, Finland, Germany, Scotland have been used in reports or articles containing national epidemiological data [14, 34, 43–48]. These registries provided better insight in incidences [43, 48], complications [45], or comorbidity [49]. Finally, data from national registries may also be used in healthcare policy making, for example, to identify therapies and treatment models that deliver for people with diabetes. Sweden, Norway, Germany, Scotland, England, and Saudi Arabia have published reports or articles containing treatment suggestions based on findings from their registry data [31, 50–54].

### Data quality

The diabetes registries from the USA and Saudi Arabia published information on the data quality assurance process. However, information about the data quality itself and the individual determinants (comparability, completeness, validity, and timeliness) was not provided [15, 22, 55–57]. In Australia, a data quality statement is published about multiple registries including the national diabetes registry; information on the quality of the registry itself or variables separately was not delivered [58]. In publications from the Finnish and the Scottish registry, the reliability of using administrative register data in the diabetes registry is discussed, yet no information is provided on quality assurance or data quality of the included variables in these registries in general [14, 59]. Publication of problems with data quality of specific variables was done by the registry of Sweden, Finland, and Scotland [28, 48, 59]. There are several parameters recorded by diabetes registries which are particularly susceptible to problems in data quality, such as the parameter diabetes classification. When the diagnosis is not according to World Health Organization recommendations or when misclassification takes place by not correctly stating the type of diabetes, this can have implications for the ability to measure quality and patient management [60–62]. Different registries use algorithms including age, treatment, and eventually clinical diabetes classification or use codes used for health reimbursement purposes [14] to overcome this issue [28, 48, 59]. This might also be the case when there is no consensus about definition criteria, such as the parameter ethnicity [63]. Examples of parameters prone to data incompleteness are PROMs [64].

## Discussion

The present study identified twelve national clinical diabetes registries spread over four different continents, mostly concentrated in the Northern part of Europe [13, 14, 57, 65–72]. Altogether, these databases comprised a total of 7,181,356 diabetic patients. All national diabetes databases harbor many parameters, yet the variety among these variables differs greatly between registries. The registries from Sweden, Denmark, Norway, Germany, Scotland, England, and the USA were able to monitor and benchmark quality against national treatment aims and identify at least one unmet medical need. All registries claim to record treatment options; Germany, Latvia, Scotland, and the USA capture glycemic control, comorbidity, and mortality to the greatest extent.

Great variety exists among diabetes registers regarding the parameters recorded. Only few parameters are registered in all databases, and the hallmark of glycemic control, HbA1c, was surprisingly not one of them. Moreover, blood pressure was not recorded in all registries, while coexistence of hypertension and diabetes significantly increases the risk of micro- and macrovascular complications and mortality [73–75], all which were also recorded inconsistently. Since clinical outcomes are the cardinal motivation to monitor diabetic patients for a longer period of time, we would have expected complications, and cardiovascular morbidity in particular, to be the cornerstone of most diabetes registries, together with HbA1c levels and insulin treatment. Alignment on what data to collect is essential to the analysis of health system performance and to inform decision-making at the national level; however, up till now there has been little collaboration among diabetes registries worldwide. Positive steps are taken by the International Diabetes Federation (IDF) that has embedded variables for screening and diagnosis, care delivery and glucose control therapy in its guidelines, as well as the International Consortium of Health Outcome Measures (ICHOM) [9, 76, 77]. Furthermore, from the perspective of the diabetic patient, patient-reported outcome measures (PROMs) are of the essence in diabetes care. Only two registries record PROMs at the moment, yet we expect the number of registries recording PROMs to rise in the next decade due to the increased medical attention for PROMs. Moreover, information on data quality and data quality assurance of recorded parameters is limited, while problems with data quality of various parameters including diabetes classification exist [28, 48]. An international consensus on methods of data validation and quality standards for parameters would also be favorable.

Most national diabetes registries aiming to improve diabetes care have accomplished better glycemic control, blood pressure management, or long-term outcomes [28, 78–82]. Yet these results need to be interpreted with caution, since it is not certain whether these changes can be attributed (only) to data from national registries or that they are driven by other policy changes, changes in treatment guidelines, publications, or trends in the population [83]. Despite the discordance and the uncertainty about the impact of diabetes care, national clinical diabetes databases have given us new insights. They have shown us that incidences of type 1 diabetes in children and young adults can be up to three times higher than expected [43]. Registries have also contributed to the discovery of unexpected complications, such as the impact of diabetes mellitus on heart failure [84], and the significant mortality in young adults with type 1 diabetes mellitus. All in all, substantial evidence exists that national diabetes registries contribute significantly to better insight and quality of diabetes care, yet its extent remains unclear [15, 16, 28, 31, 78–82]. Furthermore, several national diabetes registries have published reports or articles containing treatment suggestions based on their registry data, which only pertain to their own country [31, 50–54]. For example, in an article containing data from the Swedish diabetes registry it was suggested that diabetes screening in patients above the age of 80 should be re-evaluated and that risk factor control should be more aggressive in patients diagnosed with type 2 diabetes when below the age of 40 [50].

Regarding the future of national diabetes registries, an international consensus on essential parameters for registries would be highly desirable. Large steps could be made toward the concordance of national diabetes registries, rendering them more suitable for expansion of relevant health parameters, comparison of patient outcomes, and global improvement in diabetes care. A global standard set may be proposed based on current IDF guidelines and ICHOM set and completed with auto-antibodies in diabetes mellitus for better insights in diabetic pathophysiology, child-focused outcomes as these are currently lacking, and continuous glucose monitoring-derived data, i.e., time in range, due to its promising potential of improving glycemic control and quality of life [85]. Making health data a priority in healthcare management is vital to unlocking the potential of registries and data. Establishing the infrastructure to collect and analyze health data scattered across the health system, as well as securing the political will to apply learnings from outcomes data, is crucial.

Our study comprises many robust national registries around the globe. However, not all clinical diabetes databases worldwide were available for comments or pivotal information, rendering our review vulnerable to non-response bias. In addition, there was no insight in the registry data itself; therefore, this study was dependent on the information rendered by people involved in the diabetes registries and its published data. Moreover, registries that were not included due to other reasons such as non-national coverage might have also given us more information on the process of recording clinical diabetes outcomes throughout the world. Furthermore, only two reviewers performed the systematic review, which could make the study prone to reviewer selection bias. Finally, the definition of variables recorded in the national databases varied among registries or were lacking completely, which interfered with formulating a uniform definition for variables that encompassed the definition of all registries included. In the process toward global data exchange on diabetes care, this once again stresses the importance of standardization of outcome measures registered in diabetes registries globally. Meaningful steps have been made toward unity of structure among national diabetes registries [9, 86], but we have a long road ahead of us.

In conclusion, systematic review of national diabetes registries worldwide renders twelve registries across four continents, giving new insights on prevalence, treatment, complications, and mortality among diabetic patients. Most registries proved to monitor and improve quality of diabetes care using guidelines as a benchmark. The effects on national healthcare policy were more variable and often less clear; however, the gathered data should be able to optimize health system performance and to allocate resources and interventions where they are most needed. A globally implemented set of variables would enhance comparison and exchange of treatment and outcomes in clinical diabetes care.
